# Feasibility of a modified search, coagulation, and clipping method with and without the use of polyglycolic acid sheets and fibrin glue for preventing delayed bleeding after gastric endoscopic submucosal dissection

**DOI:** 10.1186/s12876-020-01539-x

**Published:** 2021-02-11

**Authors:** Satoshi Abiko, Soichiro Oda, Akimitsu Meno, Akane Shido, Sonoe Yoshida, Ayumu Yoshikawa, Kazuaki Harada, Naoki Kawagishi, Itsuki Sano, Hisashi Oda, Takuto Miyagishima

**Affiliations:** 1 Department of Gastroenterology, Kushiro Rosai Hospital, Kushiro, Japan; 2 Department of Gastroenterology and Hepatology, Hakodate Municipal Hospital, Hakodate, Japan

**Keywords:** Delayed bleeding, Gastric endoscopic submucosal dissection, Polyglycolic acid sheets

## Abstract

**Background:**

Methods have been developed for preventing delayed bleeding (DB) after gastric endoscopic submucosal dissection (GESD). However, none of the methods can completely prevent DB. We hypothesized that DB could be prevented by a modified search, coagulation, and clipping (MSCC) method for patients at low risk for DB and by combining the use of polyglycolic acid sheets and fibrin glue with the MSCC method (PMSCC method) for patients at high risk for DB (antibleeding [ABI] strategy). This study assessed the technical feasibility of this novel strategy.

**Method:**

We investigated 123 lesions in 121 consecutive patients who underwent GESD in Kushiro Rosai Hospital between April 2018 and January 2020. The decision for continuation or cessation of antithrombotic agents was based on the Guidelines for Gastroenterological Endoscopy in Patients Undergoing Antithrombotic Treatment.

**Results:**

Oral antithrombotic agents were administered to 28 patients (22.8%). The en bloc R0 resection rate was 98.4%. The MSCC method and the PMSCC method for preventing DB were performed in 114 and 9 lesions, respectively. The median time of the MSCC method was 16 min, and the median speed (the resection area divided by the time of method used) was 3.6 cm^2^/10 min. The median time of the PMSCC method was 59 min, and the median speed was 1.3 cm^2^/10 min. The only delayed procedural adverse event was DB in 1 (0.8%) of the 123 lesions.

**Conclusions:**

The ABI strategy is feasible for preventing DB both in patients at low risk and in those at high risk for DB after GESD, whereas the PMSCC method may be necessary for reduction of time.

## Background

Gastric cancer is the fifth most common cancer worldwide, and endoscopists in Eastern Asian countries detect 50–70% of gastric cancers at an early stage [1–4]. Gastric endoscopic submucosal dissection (GESD) is a widespread technique for treatment of gastric epithelial neoplasms [4, 5]. However, post-ESD bleeding is a serious complication after GESD, with a reported incidence of 3–5.5% [6–11]. This complication sometimes results in shock requiring transfusion. A safe, simple, and cost-efficient method of preventing post-ESD bleeding should be developed. Post-ESD coagulation (PEC) has been used in many hospitals to prevent post-ESD bleeding [6]. The search, coagulation, and clipping (SCC) method has been reported to be much better than the PEC method for preventing post-ESD bleeding [12]. However, the SCC method does not completely prevent post-ESD bleeding. Another approach combines the use of polyglycolic acid (PGA) sheets and fibrin glue to reduce the risk of DB after GESD [13]. However, a multicenter, prospective, randomized controlled study showed that PGA sheets alone were not effective in preventing DB in patients at high risk for such bleeding [14]. We have devised a new method of preventing DB after GESD by combining the use of PGA sheets and fibrin glue with a modified SCC method [15]. We hypothesized that DB could be prevented by a modified SCC method (MSCC method) for patients at low risk for DB and by combining the use of PGA sheets and fibrin glue with the MSCC method (PMSCC method) for patients at high risk for DB (antibleeding [ABI] strategy). This study assessed the technical feasibility of this novel strategy.

## Methods

### Study design

This study was a retrospective case series.

### Subjects

We investigated 123 lesions in 121 consecutive patients who underwent GESD in the Department of Gastroenterology at Kushiro Rosai Hospital between April 2018 and January 2020. The surgeries were performed by an endoscopist (S.A.) and trainees with experience of fewer than 30 ESD procedures in humans. All of the procedures were tutored by one endoscopist (S.A.). Until March 2018, ESD was performed in 169 patients (pharynx in 1 patient, esophagus in 16 patients, stomach in 128 patients, and large intestine in 24 patients) under the guidance of tutors at other institutions. Perforation and delayed perforation did not occur in any cases, and DB occurred in four cases (3.1%, ESD of the stomach). From April 2018, S.A. was the only endoscopist for ESD at our institution. Patients at high risk for DB were classified as patients being administered direct oral anticoagulants (DOACs) or warfarin, patients on dialysis, and patients who had received heparin replacement. Other patients were classified as patients at low risk for DB. We evaluated the number of en bloc R0 resections; the number of curative resections; length of the tumor; length of the resected specimen; resection area; resection time; resection speed; method of preventing DB; number of clips used for the MSCC method; time and speed of the PEC, MSCC, and PMSCC methods with and without the use of second-look endoscopy and vonoprazan; the number of procedures at the second-look or follow-up endoscopy; and procedural and delayed procedural adverse events, including perforation during ESD, delayed perforation, DB, and post-ESD stenosis. En bloc R0 resection was defined as resection of the tumor in a single piece with tumor-free lateral and vertical margins. Resection time was the duration from the first injection until achievement of complete resection. Resection speed was defined as the resection area divided by the resection time (cm^2^/h). The resection area was regarded as being approximately oval in shape. The time of the PEC, MSCC, and PMSCC methods was defined as the interval from insertion of the first device for the prevention of DB until completion of the method. The speed of the PEC, MSCC, and PMSCC methods was defined as the resection area divided by 10× the time of the PEC, MSCC, and PMSCC methods (cm^2^/10 min). Perforation was defined as the creation of an immediately recognized hole in the gastric wall. Delayed perforation was defined as the presence of free air on abdominal computed tomography or X-ray after completion of the procedure in patients without perforation during ESD and no symptoms of peritoneal irritation after ESD. DB was defined as bleeding requiring emergency endoscopic hemostasis or transfusion or the presence of hemoglobin loss ≥ 2 g/dL following ESD [16]. Delayed procedural adverse events were assessed for 30 days post-ESD. Continuation or cessation of antithrombotic agents was determined according to the Guidelines for Gastroenterological Endoscopy in Patients Undergoing Antithrombotic Treatment [17, 18]. We examined ABO blood type because it has been reported that blood type O may be less likely to clot than other types [19].

### ESD method and management after ESD

GIF-H290Z (Olympus Optical, Tokyo, Japan) and a needle knife were used for assessing the lesion margin and marking around the lesion. ESD was performed with the patient under conscious sedation using a single-channel gastrointestinal endoscope with a transparent attachment hood fitted to the tip (GIF-Q260J; Olympus Optical). When difficulties were observed, we used another twin-channel gastrointestinal endoscope (GIF-2TQ260M; Olympus Optical). The GIF-2TQ260M endoscope has a multi-bending and a water jet function. Hyaluronic acid solution was injected into the submucosal layer before mucosal and submucosal cutting. After injection, we mainly performed mucosal cutting with a needle knife and dissection beneath the lesion using an IT knife-2 (Olympus Optical). When difficulties were observed, we used a hook knife (Olympus Optical), flush knife (Fujifilm Optical), or clutch cutter (Fujifilm Optical). We used a VIO 200D electrosurgical generator (ERBE Elektromedizin, Tübingen, Germany). Hemorrhage was controlled using hemostatic forceps such as Coagrasper (Olympus Optical monopolar hot hemostasis forceps) for the upper digestive tract.

After ESD, a proton-pump inhibitor (omeprazole 20 mg twice a day) was intravenously injected. The patient’s doctor in the ward rarely ordered a second-look endoscopy to be performed on postoperative day (POD) 1. If there were no problems, oral food intake was started on POD 2. Then an oral proton-pump inhibitor (esomeprazole 20 mg/day, lansoprazole 30 mg/day, or rabeprazole 20 mg/day) or vonoprazan (20 mg/day) was administered for a minimum of 8 weeks. Sodium alginate (60 mL/day) and aluminum hydroxide gel, magnesium hydroxide (160 mL/day) were administered for a minimum of 3 days. Before the patient was discharged from the hospital, follow-up endoscopy was performed on POD 5–7.

### Methods for prevention of DB

We previously described a new method combining the use of PGA sheets and fibrin glue with a modified SCC method for preventing DB after GESD [15]. First, a coagulation procedure was performed after resection of the lesion, mainly in the vessels at the margin of the ulcer base. Then, the perforator vessels emerging between the muscle layers were actively sought and clipped using short hemoclips (HX-610-135S, Olympus Optical). Because perforator vessels may also be present in the carbonized areas of the ulcer base, clipping was also actively performed in such areas; this constituted the modification of the SCC method. For patients at low risk for DB, we ended with this procedure. For patients at high risk for DB, we moved on to the next procedure. In the next procedure, several large and small PGA sheets were placed (based on the size of the ulcer base), using methods described by Kobayashi et al. [20] and Takimoto et al. [21]. Finally, fibrin glue was sprayed. All these steps constituted the polyglycolic acid sheets, fibrin glue, and modified search, coagulation, and clipping (PMSCC) method.

### Pathological assessment of resected specimens

The resected specimens from the stomach were cut into longitudinal slices 2 to 3 mm in width that were embedded in paraffin. The slices were stained with hematoxylin–eosin and examined microscopically. Curative resection of adenocarcinoma was previously described by the Japanese Gastric Cancer Association [22].

### Ethical approval

The study was conducted in accordance with the rules and regulations of the Kushiro Rosai Hospital Institutional Review Board (study registration number: 19233) and conformed to the requirements of the Declaration of Helsinki. Written informed consent was obtained from all study subjects.

### Statistical analysis

All statistical analyses were performed with EZR (Saitama Medical Center, Jichi Medical University, Saitama, Japan), which is a graphical user interface for R (The R Foundation for Statistical Computing, Vienna, Austria). More precisely, EZR is a modified version of R commander designed to add statistical functions frequently used in biostatistics [23]. Continuous and nonparametric variables were expressed as medians with 25th and 75th percentile values. The Mann–Whitney U test was used for comparisons of continuous and nonparametric variables, and the Fisher exact test was used for comparisons of categorical variables. Differences were considered statistically significant at a *P* value < 0.05.

## Results

The median age of the subjects was 73 (interquartile range [IQR], 69–79) years, and the male/female ratio was 98/25. Oral antithrombotic agents were administered to 28 patients (22.8%). The lesion sites were upper (*n* = 16), middle (*n* = 59), and lower (*n* = 48) portions of the stomach. The median length of the long tumor axis was 1.3 (IQR 0.7–2.0) cm, the median resected specimen length was 3.0 (IQR 2.5–4.1) cm, and the median resection area was 5.5 (IQR 3.5–9.4) cm^2^ (Table 1). The en bloc R0 resection rate was 98.4%, and the curative resection rate was 87.0%. The median resection time was 91 (IQR 55–137) min and the median resection speed was 4.5 (IQR 2.5–5.4) cm^2^/h. The MSCC and PMSCC methods for preventing DB were performed in 114 and 9 lesions, respectively. The median time of the MSCC method was 16 (IQR 8–23) min, and the median speed was 3.6 (IQR 2.8–4.1) cm^2^/10 min. The median time of the PMSCC method was 59 (IQR 44–63), min and the median speed was 1.3 (IQR 1.2–2.4) cm^2^/10 min. The only delayed procedural adverse event was DB in 1 (0.8%) of the 123 lesions. There were no significant differences in the results and clinical course of GESD between patients treated with MSCC and those treated with PMSCC (Table 2).

**Table 1:** Characteristics of patients and lesions

Characteristic	Value
Age	
Median (IQR), years	73 (69–79)
Sex	
Male/female	98/25
Oral antithrombotic agents, *n* (%)	
None	95 (77.2)
Warfarin	3 (2.4)
DOAC	3 (2.4)
DAPT	1 (0.8)
Clopidogrel	3 (2.4)
Aspirin	7 (5.7)
Aspirin + others	1 (0.8)
Cilostazol	2 (1.6)
Others	8 (6.5)
Dialysis, *n* (%)	2 (1.6)
Heparin replacement, *n* (%)	1 (0.8)
Lesion site, *n* (%)	
Upper	16 (13.0)
Middle	59 (48.0)
Lower	48 (39.0)
Length of resected specimen	
Median (IQR), cm	3.0 (2.5–4.1)
Resection area	
Median (IQR), cm^2^	5.5 (3.5–9.4)
Length of tumor	
Median (IQR), cm	1.3 (0.7–2.0)
Pathological diagnosis, *n* (%)	
Adenocarcinoma	106 (86.2)
Adenoma	14 (11.4)
Others	3 (2.4)
Depth, *n* (%)	
Intramucosa	99 (80.5)
Submucosa	24 (19.5)
Macroscopic type, *n* (%)	
Depressed	52 (42.3)
Nondepressed	71 (57.7)
Submucosal fibrosis, *n* (%)	
Negative	107 (87.0)
Positive	16 (13.0)
Blood type, *n* (%)	
O	40 (32.5)
A	37 (30.1)
B	35 (28.5)
AB	11 (8.9)

*DAPT* dual antiplatelet therapy, *DOAC* direct oral anticoagulant, *IQR* interquartile range, *PMSCC* polyglycolic acid sheets and fibrin glue combined with the modified search, coagulation, and clipping method

**Table 2:** Results and clinical course of gastric ESD

Characteristic	Total (*n* = 123)	MSCC (*n* = 114)	PMSCC (*n* = 9)	*P* value
En bloc R0 resection, *n* (%)	121/123 (98.4)	112/114 (98.2)	9/9 (100.0)	1.0000
VM1, *n* (%)	2 (1.6)	2 (1.8)	0 (0)	1.0000
HM1, *n* (%)	0 (0)	0 (0)	0 (0)	
Curative resection, *n* (%)	107/123 (87.0)	86/100 (86.0)	7/9 (77.7)	0.3312
Resection time (min), median (IQR)	91 (55–137)	88 (54–133)	100 (74–186)	0.3486
Resection speed (cm^2^/h), median (IQR)	4.5 (2.6–6.2)	4.5 (2.6–6.2)	4.0 (2.2–5.2)	0.7819
Surgeon, S.A. alone/others	84/39	77/37	7/2	0.7177
Time of PEC method (min), median (IQR)	7 (5–11)	7 (4–11)	11 (5–16)	0.1060
Speed of PEC method (cm^2^/10 min), median (IQR)	7.5 (4.9–12.7)	7.5 (4.9–12.8)	6.9 (4.5–11.4)	0.7449
Time of MSCC method (min), median (IQR)	16 (8–23)	16 (8–22)	26 (8–30)	0.2574
Speed of MSCC method (cm^2^/10 min), median (IQR)	4.0 (2.7–7.1)	4.0 (2.7–6.9)	4.3 (2.8–7.7)	0.6656
Number of clips used for MSCC method, *n*, median (IQR)	4 (1–7)	4 (1–7)	4 (4–8)	0.3884
Time of PMSCC method^a^ (min), median (IQR)			59 (44–63)	
Speed of PMSCC method^a^ (cm^2^/10 min), median (IQR)			1.3 (1.2–2.4)	
Second-look endoscopy, *n* (%)	15 (12.9)	13 (12.9)	2 (22.2)	0.3015
Vonoprazan, *n* (%)	99 (80.5)	90 (78.9)	9 (100.0)	0.2032
Perforation during ESD, *n* (%)	0 (0)	0 (0)	0 (0)	
Delayed perforation, *n* (%)	0 (0)	0 (0)	0 (0)	
Delayed bleeding, *n* (%)	1 (0.8)	1 (0.9)	0 (0)	1.0000
Post-ESD stenosis, *n* (%)	0 (0)	0 (0)	0 (0)	

*ESD* endoscopic submucosal dissection, *HM1* horizontal margin positive, *IQR* interquartile range, *MSCC* modified search, coagulation, and clipping method, *PEC* post-ESD coagulation, *PMSCC* polyglycolic acid sheets and fibrin glue combined with the modified search, coagulation, and clipping method, *VM1* vertical margin positive

^a^Only the nine patients in whom PMSCC was performed are included

Table 3 shows details of the nine patients in whom the PMSCC method was used. The PMSCC method was used in nine patients at high risk for DB for the following reasons: heparin replacement (one patient), hemodialysis (two patients), direct oral anticoagulant administration (three patients), and oral warfarin administration (three patients). DB did not occur in any of the nine patients (Table 3).

**Table 3:** Details of the nine patients in whom the PMSCC method was used

Patient	Age (yr)	Sex	Lesion site	Length of resected specimen (cm)	Oral antithrombotic agent	Dialysis	Heparin replacement	Vonoprazan	Blood type
1	78	M	Lower	4.3	Aspirin	−	**+**	**+**	A
2	85	M	Lower	4.3	Nicorandil	**+**	−	**+**	B
3	72	M	Middle	2.2	Warfarin	−	−	**+**	A
4	74	M	Middle	4.8	DOAC	−	−	**+**	A
5	66	M	Upper	4.1	DOAC	−	−	**+**	A
6	66	M	Middle	7.0	−	**+**	−	**+**	O
7	70	M	Middle	1.9	Warfarin	−	−	**+**	O
8	74	M	Middle	4.0	Warfarin	−	−	**+**	B
9	61	M	Middle	3.1	DOAC **+ **others	−	−	**+**	B

*DOAC* direct oral anticoagulant, *PMSCC* polyglycolic acid sheets and fibrin glue. Combined with the modified search, coagulation, and clipping method

## Discussion

In this study, we investigated the technical feasibility of this novel strategy and found that DB occurred in only one patient, the time of the MSCC method was acceptable, the use of the PMSCC method prevented DB in patients at high risk for DB after GESD, and the time of the PMSCC method was not acceptable.

DB after ESD is a serious complication and should be prevented [7]. The incidence of DB after GESD has been reported to be 3–5.5% [6–11], and the incidence of DB in patients receiving antithrombotic therapy after ESD has been reported to be 21–38% [24–27]. Methods for the prevention of DB after ESD were reported by Takizawa et al. (the incidence of DB in all patients was 3.1%) [6], Mukai et al. (the incidence of DB in all patients was 1.3%) [28], Azumi et al. (the incidence of DB in all patients was 2.6%) [12], and Kawata et al. (the incidence of DB in patients receiving continued antithrombotic therapy was 5.8%) [13], but no methods completely prevent DB after ESD. We hypothesized that DB could be prevented by combining the use of PGA sheets and fibrin glue with the MSCC method (the PMSCC method) in patients at high risk for DB [15] compared with other studies. In this study, the incidence of DB in all patients was 0.8%, and the incidence of DB in patients receiving antithrombotic therapy was 3.6%. Thus, the ABI strategy may be useful for preventing DB in patients after GESD.

In recent years, attempts have been made to endoscopically suture the ulcer base for prevention of DB in patients after GESD. However, the stomach has a thick wall, and it is difficult for the sutures to be retained. It appears that the sutures can only be retained for a few days [29]. A recent study reported that the retention of the sutures was gradually improved, but the incidence of DB was 10% [30]. With current endoscopic techniques, complete suture of the ulcer base in the stomach appears to be difficult. Therefore, the ABI strategy is a reasonable method for preventing DB in patients after GESD.

The region in which bleeding occurs in ESD is often carbonized. When a region becomes carbonized, it is difficult to recognize exposed blood vessels, and subsequent DB prevention measures may be neglected. With our method, clipping was actively performed at carbonized sites. If bleeding does not occur during ESD, exposed blood vessels can easily be recognized, which may be important for preventing DB.

With regard to the merits of the PMSCC method, although bleeding may be observed from perforator vessels in areas where the PGA sheets are peeled off [14], we believe that if perforator vessels at the base of ulcers are clipped in advance, such DB may be prevented. With regard to the limitations of the PMSCC method, it can be difficult to identify and clip all perforator vessels, and depending on the meal, the PGA seat may be peeled off. It may be necessary for the patient to fast after application of the PMSCC method in patients at very high risk for DB after resuming antithrombotic agents.

DOACs are associated with gastrointestinal bleeding, and it has been hypothesized that nonabsorbed, active anticoagulant agents within the gastrointestinal tract induce bleeding of fragile mucosal defects [31]. In this study, fragile mucosal defects were protected by PGA sheets. Prevention of DOAC-related DB by the PMSCC method may be possible.

Hatta et al. derived and externally validated a prediction model for DB after GESD, with good performance metrics [32]. We were able to identify patients with a risk of DB after GESD but did not know how manage them. It is not practical to use the complicated PMSCC method in all patients. The simple MSCC method should be used in low-risk patients, and the complicated PMSCC method should only be used in high-risk patients. In the future, we may decide which method to use depending on the number of DB risk factors.

The procedure time of the PMSCC method was not acceptable. However, we are now developing a new technique to shorten the procedure time of PMSCC. We hope to report the results soon.

The study had three main limitations. There was only one principal surgeon, the sample size was small, and the study design was retrospective. Future prospective studies with larger sample sizes and randomized controlled trials are needed to evaluate the utility of our method.

## Conclusions

The ABI strategy is feasible for preventing DB in patients at low risk or at high risk for DB after GESD, whereas the PMSCC method may be necessary to reduce the time.

## Data Availability

From the corresponding author on reasonable request.

## Abbreviations

DB: Delayed bleeding
GESD: Gastric endoscopic submucosal dissection
MSCC: Modified the search, coagulation, and clipping
ABI: Anti-bleeding
PEC: Post-ESD coagulation
SCC: Search, coagulation, and clipping
ESD: Endoscopic submucosal dissection
PGA: Polyglycolic acid
POD: Postoperative day
IQR: Interquartile range
GI: Gastrointestinal
